# Large-Scale Analysis of Antimicrobial Activities in Relation to Amphipathicity and Charge Reveals Novel Characterization of Antimicrobial Peptides

**DOI:** 10.3390/molecules22112037

**Published:** 2017-11-22

**Authors:** Chien-Kuo Wang, Ling-Yi Shih, Kuan Y. Chang

**Affiliations:** 1 Department of Biotechnology, Asia University, Taichung 413, Taiwan; ck@asia.edu.tw; 2 Computational Biology Laboratory, Department of Computer Science & Engineering, National Taiwan Ocean University, Keelung 202, Taiwan; 10257001@ntou.edu.tw

**Keywords:** antimicrobial peptides, antimicrobial activities, amphipathicity, net charge

## Abstract

It has been unclear to which antimicrobial activities (e.g., anti-gram-positive bacterial, anti-gram-negative bacterial, antifungal, antiparasitic, and antiviral activities) of antimicrobial peptides (AMPs) a given physiochemical property matters most. This is the first computational study using large-scale AMPs to examine the relationships between antimicrobial activities and two major physiochemical properties of AMPs—amphipathicity and net charge. The results showed that among all kinds of antimicrobial activities, amphipathicity and net charge best differentiated between AMPs with and without anti-gram-negative bacterial activities. In terms of amphipathicity and charge, all the AMPs whose activities were significantly associated with amphipathicity and net charge were alike except those with anti-gram-positive bacterial activities. Furthermore, the higher the amphipathic value, the greater the proportion of AMPs possessing both antibacterial and antifungal activities. This dose–response-like pattern suggests a possible causal relationship—dual antibacterial and antifungal activities of AMPs may be attributable to amphipathicity. These novel findings could be useful for identifying potent AMPs computationally.

## 1. Introduction

A large-scale analysis of the biological activities of antimicrobial peptides (AMPs) has been carried out. AMPs can either kill or inhibit the growth of microbial organisms such as bacteria, fungi, parasites, and viruses. Not all AMPs are alike. Some such as moronecidin and melittin exhibit broad-spectrum antimicrobial activities with antibacterial, antifungal, antiparasitic, and antiviral effects [1,2]; some possess limited antimicrobial effects. For example, ganodermin is an anti-fungal peptide [3]; psalmopeotoxin-2 is only known for antimalarial activities [4]; serracin P is a bacteriocin for gram-negative bacteria [5]. Until now, which property makes some AMPs effective against specific types of microbes was unclear. In fact, large-scale bioinformatic studies on AMP physicochemical properties have been done [6,7,8,9]. However, none of these large-scale studies extensively examined the relationships between the physicochemical properties and antimicrobial activities of AMPs.

Large-scale data can reveal new insights on AMPs. AMP biology began from a few peptides. Small-scale data are excellent at offering a quick and preliminary analysis. However, observation and deduction drawn from limited data suffer over-generalization and can easily succumb to logical fallacies. In contrast, large-scale data overcome this limitation by providing a big picture. For example, small-scale and limited data such as magainin 2 peptide analogs showed that an increase in amphipathicity promoted an increase in antibacterial activities [10], but extensive analyses with more data suggested that perfect amphipathicity was unnecessary for potent antimicrobial activities [11].

It remained a mystery whether significant propensities exist behind the complex relations between physicochemical properties and antimicrobial activities of AMPs. Which antimicrobial activities of AMPs (e.g., anti-gram-positive bacterial, anti-gram-negative bacterial, antifungal, antiparasitic, or antiviral activities) a given physicochemical property matters most to is waiting to be unraveled by large-scale AMP studies. In fact, large-scale data can uncover trends and correlations, but they are difficult to acquire. It took more than half a century to have thousands of AMPs experimentally validated since the first documented AMP in the early 20th century [7]. However, the general trends which neither depend on one special case nor on a handful of artificial peptide analogs should only be resolved by large-scale AMPs. This is especially true when the underlying relations are complex.

Besides, additional unwanted and uncontrolled variables can be introduced into the peptide analog validation to worsen and complicate these analyses. For example, modifying the amphipathicity of a peptide often simultaneously changes other charge-related properties (e.g., net charge). To demonstrate the effectiveness of amphipathicity of an AMP in relation to a specific antimicrobial activity, experimentally abolishing amphipathicity of its few derived artificial peptides was typically done, while accidentally altering their net charges, causing ambiguity in deducing the exact role of the original factor regardless of whether the specific activity of the AMP vanishes. In this case, it would be logical to ask whether amphipathicity (controlled variable), net charge (additional uncontrolled variable), or the combination of amphipathicity and net charge (interaction) matter to any specific antimicrobial activities (dependent variable). However, such thorough investigations are lacking, as it is difficult for individuals to undergo a great number of extra experiments. Fortunately, due to the outstanding statistical power, large-scale AMPs which neither need extra experiments nor unwanted variables could provide clearer and more accurate assessments than limited AMPs.

In this study, we investigated the associations between specific antimicrobial activities and the two major physicochemical properties of AMPs: net charge and amphipathicity. Using the large AMP peptidome available to us with more than three thousand experimentally-validated AMPs [7], a thorough examination between the two physicochemical properties and antimicrobial activities was performed. To avoid bias, redundant and highly similar AMP sequences were removed from our analysis. Thanks to the large size of the AMPs, novel characterization of AMPs was recognized—AMPs with anti-gram-negative bacterial activities demonstrated the strongest propensities toward high amphipathicity and positive net charge; AMPs with anti-gram-positive bacterial activities might be one of a kind; the dose–response-like pattern occurred between amphipathicity and AMPs possessing dual antibacterial and antifungal activities.

## 2. Results

### 2.1. AMPs with Anti-Gram-Negative Bacterial Activities Strongly Favor Amphipathicity and Cationic Charge

Table 1 compares the presence and absence of specific antimicrobial activities of AMPs in relation to amphipathicity or net charge. A one-sided Mann–Whitney U test was chosen to analyze the results because normality tests indicated that the amphipathic or charge distributions were not a standard normal distribution. We examined 31 hypotheses—that is, all the combinations of five specific antimicrobial activities such as anti-gram-positive bacterial, anti-gram-negative bacterial, antifungal, antiparasitic, and antiviral activities except null activities—and listed those—only seven of them—which passed the U tests, adjusting by the Bonferroni correction for multiple comparisons with the significance level of α = 0.01. In addition, further examinations of the AMPs with these seven specific antimicrobial activities were done to determine any significant differences among them. Only two out of the 21 comparisons were found significant, shown at the bottom of Table 1.

Table 1 demonstrates that the amphipathiciy and net charge between the AMPs with and without anti-gram-negative bacterial activities differ the most compared to those with any other antimicrobial activities. The largest differences of amphipathicity and net charge between those with and without anti-gram-negative bacterial activities were 0.130 (μH) and 2 (Q), respectively. Table 1 also shows that the anti-gram-negative bacterial, anti-gram-positive bacterial, or anti-fungal AMPs had higher net charge and amphipathic values than their counterparts, although the differences of amphipathicity between anti-fungal peptides and non-anti-fungal peptides were not sufficient to survive the Bonferroni correction. No significant differences were found between antiviral peptides and non-antiviral peptides. The same was true for antiparasitic peptides.

### 2.2. AMPs with Anti-Gram-Positive Bacterial Activities Differ from Other AMPs in Amphipathicity and Net Charge

Table 1 also demonstrates that the AMPs with anti-gram-positive bacterial activities are least cationically charged and least amphiphilic among all the significant relationships. The AMPs which possess at least anti-gram-positive bacterial activities were found to be significantly different from those which possess at least dual activities, anti-gram-negative bacterial and anti-fungal activities, or triple activities (the coexistence of anti-gram-positive bacterial, anti-gram-negative bacterial, and anti-fungal activities). In fact, amphipathicity and positive charge tended to be higher among the AMPs with broader antimicrobial activities, although most of their differences failed to pass the Bonferroni correction. Only the AMPs with anti-gram-negative bacterial and anti-fungal activities or anti-gram-positive bacterial, anti-gram-negative bacterial, and anti-fungal activities were found to have significantly higher amphipathicity and charge compared to those with anti-gram-positive bacterial activities. For example, thuricin CD with 0.420 (μH) and −1 (Q), known only for its anti-gram-positive bacterial activities, has lower amphipathicity and net charge than pilosulin 5 with 0.799 (μH) and 4 (Q), known for its anti-gram-negative bacterial and antifungal activities [7].

### 2.3. Trends of Antimicrobial Activities of AMPs in Terms of Amphipathicity and Net Charge

**Figure 1:**
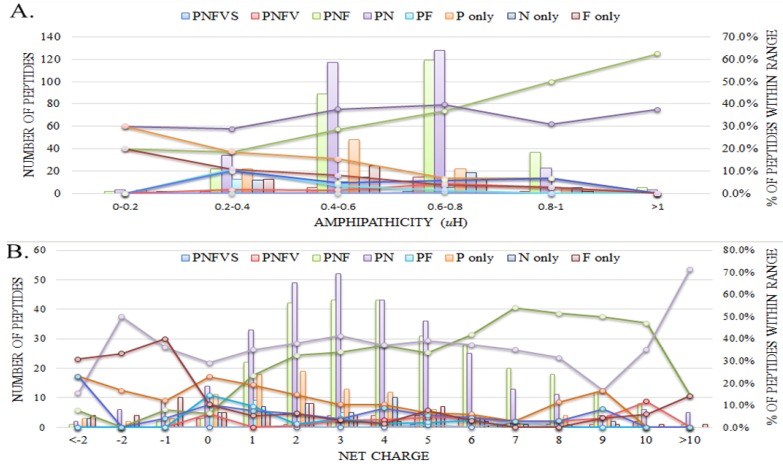
Distributions of antimicrobial activities of AMPs with respect to (**A**) amphipathicity and (**B**) net charge. The distribution of the AMPs with specific antimicrobial activities is displayed using a histogram on the left axis, and the percentages of the AMPs with specific antimicrobial activities in the specific range are demonstrated using a line chart on the right axis. Abbreviations: P: anti-gram-positive bacteria; N: anti-gram-negative bacteria; F: anti-fungi; V: anti-viruses; S: anti-parasites.

Figure 1A reveals the distribution of antimicrobial activities of AMPs in relation to amphipathicity. Peptides with all kinds of antimicrobial activities generally occurred in the range of amphipathic value from 0.4 to 0.8. As the amphipathic values increased, the number of the AMPs generally decreased. However, the proportion of peptides with both antibacterial (anti-gram-positive and anti-gram-negative) and antifungal activities steadily increased. Once the amphipathic values exceeded 0.8, AMPs which possessed both antibacterial and antifungal activities became dominant. Approximately half of the AMPs with high amphipathic values were those with both antibacterial and antifungal activities.

Figure 1B exhibits the distribution of antimicrobial activities of AMPs in relation to net charge. Net charge can be positive or negative, but neither the distribution of net charge of the AMPs nor the distribution of net charge of the percent of peptides within a specific range was symmetric about the axis of zero net charge. These asymmetric distributions had noticeable characters. For example, about one fourth of the AMPs with negative net charge only had antifungal activities. We also observed similar results to what have been reported before—four out of five AMPs had a positive net charge [12], about half between +2 and +5.

### 2.4. The Higher Amphipathicity, the Greater the Proportion of AMPs Possessing Antibacterial/Antifungal Activities

Figure 1 shows the general trend of AMPs. Except for the categories of negative net charge, more AMPs with antibacterial activities and antibacterial/antifungal activities were recorded than others in all the categories of amphipathicity and net charge. Figure 1A reveals a clear AMP trend showing that high amphipathicity more likely happens to be the AMPs presented with antibacterial/antifungal activities than those with antibacterial activities or any others. Figure 1B shared a slightly similar trend—as the net positive charge increased, the proportion of peptides with both antibacterial and antifungal activities generally increased. However, unlike amphipathicity in Figure 1A, the peptides with both antibacterial and antifungal activities failed to keep leading at the extreme cases of net charges over +10. Other than the AMPs with antibacterial/antifungal activities, we also observed that neither the number nor the portion of those with with broader antimicrobial activities increased as the amphipathic values or net positive charges increased.

Amphipathicity and net charge are conceptually different, but Figure 1A with amphipathicity and Figure 1B with net charge were similar in some way. Thus our further analysis showed that with respect to AMPs, amphipathicity and net charge were different but modestly positively correlated, with Pearson’s correlation = 0.51.

## 3. Discussion

To our knowledge, this was the first study to thoroughly examine the antimicrobial activities of AMPs with respect to their physiochemical properties. The study demonstrated that AMPs with anti-gram-positive bacterial, anti-gram-negative bacterial, or anti-fungal activities generally possessed higher net charge and amphipathic values than their counterparts. Our results indicated that amphipathicity and cationic charge should play the most critical roles in anti-gram-negative bacterial activities than in the other antimicrobial activities of AMPs due to the largest significant differences and the smallest *p*-value, which is a novel finding that could only be demonstrated by large-scale AMP data. One explanation for this association may be that the outer membrane of the gram-negative bacterial cell wall, containing negatively-charged lipopolysaccharide, may well captivate positively-charged amphipathic AMPs. Alternatively, because these highly-charged and amphipathic anti-gram-negative bacterial peptides are similar to cell-penetrating peptides [13], they can easily penetrate the outer membrane of gram-negative bacteria to interact with the relatively weaker peptidoglycan in the cell wall, causing the rupture of gram-negative bacteria. By any means, to recognize or design peptides which possess anti-gram-negative bacterial activities, special attention should be paid to amphipathicity and cationic charge.

Few interesting not-previously-reported phenomena which may have biological meanings were observed in this large-scale AMP study. All the AMPs whose activities were significantly associated with amphipathicity and net charge were alike, except for those with anti-gram-positive bacterial activities. Our results indicated that the AMPs with anti-gram-negative bacterial activities and those with antifungal activities were closer in terms of amphipathicity and net charge distributions than those with anti-gram-positive bacterial activities. Besides, an unusually large portion of the AMPs with negative net charge were antifungal only, indicating that some antifungal peptides would fundamentally differ from antibacterial peptides or others. These results reflect the distinctions between bacteria and fungi, such as cell wall. Rather than the peptidoglycan make-up of the bacterial cell wall, the fungal cell wall is composed mostly of chitin, β-glucan, and mannoprotein.

This study was also the first to demonstrate a dose–response-like trend between amphipathicity and dual antibacterial and antifungal activities. As amphipathicity and net charge increased, antibacterial activities and antifungal activities seemed to tangle more closely. Particularly, the higher the amphipathic value, the higher the proportion of AMPs with these dual activities. This dose–response relationship suggests a possible causal relationship, supporting that amphipathicity might be a cause of the dual antimicrobial activities of AMPs.

This study demonstrated that amphipathicity and net charge differentiate between AMPs with and without anti-gram-negative bacterial activities the best. However, why amphipathicity and net charge matter the most in the anti-gram-negative bacterial activities is unclear. The precise mechanism of how amphipathicity and charge assist the AMP action against bacteria and fungi is under debate. Although the nature of amphipathic and cationic AMPs does obey the models of abruption of microbial cytoplasmic membrane [14], it does not reject the possibilities of interacting electrostatically with microbial DNAs, proteins, or lipids to inhibit the growth of microbes.

Neither amphipathicity nor net charge were found to significantly associate with antiviral or antiparasitic peptides in this study. Any AMPs involved with viruses or parasites showed no distinctions, although those with concurrent antibacterial, antifungal, and antiviral activities which did pass the initial screen failed the Bonferroni correction. Additional data might help to affirm whether amphipathicity or charge significantly associates with the coexistence of antibacterial, antifungal, and antiviral activities of AMPs.

## 4. Materials and Methods

Five major biological activities of AMPs were investigated in this study, including anti-gram-positive bacterial, anti-gram-negative bacterial, anti-fungal, anti-viral, and anti-parasitic activities. Other activities such as anti-cancer were ignored. The compiled information was obtained online from A Database of Anti-Microbial peptides (ADAM http://bioinformatics.cs.ntou.edu.tw/ADAM/download.html) [7].

### 4.1. Non-Redundant Experimentally-Validated AMPs

The data were collected by following the procedure which was previously described in Chang et al. (2015) with some modification; 3152 non-redundant experimentally-validated AMPs were obtained from ADAM [7]. Among these validated AMPs, 2113 AMPs without any ambiguous amino acids such as “B”, “U”, “X”, and “Z” whose lengths range from 7 to 40 amino acids long were investigated. In our previous study [15], we identified that some AMPs would nest on other longer sequences. Here 138 nested AMP families were directly extracted from this dataset, and each nested family left one core sequence behind. To eliminate highly similar sequences in the dataset, Cluster Database at High Identity with Tolerance (CD-HIT) [16] was utilized to cluster the sequences over 70% identity into a family. By default, CD-HIT picked the longest sequence to represent the highly similar sequence family. However, to emphasize the minimal functional unit, the shortest AMP in the family—with preference to the nested AMP cores—was selected instead; 1050 AMPs were thus extracted. Then, 130 out of these 1050 AMPs were discarded because the information about what kind of microbes they acted against was lacking. Eventually, there were 920 non-redundant experimentally-validated AMPs in the final dataset.

### 4.2. AMP Amphipathicity

Amphipathicity or amphiphilicity is the spatial coexistence of hydrophobicity on one side and hydrophilicity on the opposite side of an object. The hydrophobic moment μ of a peptide, which measures the strength of amphipathicity, is calculated using the following formula [17]:

(1) $$u=\sqrt{[\sum_{n=1}^{N}H_{n}\sin\left(\delta n\right){]}^{2}+[\sum_{n=1}^{N}H_{n}\cos\left(\delta n\right){]}^{2}}$$

where *n* is the *n*th amino acid of the peptide, $H_{n}$ is the hydrophobic value of the *n*th amino acid, and δn is the degree of the *n*th amino acid projected on a two-dimensional circle. The hydrophobic moment μ is a nonnegative real number. The larger the μ, the stronger the amphipathicity of the peptide. That is, one side being hydrophobic and the other side being hydrophilic becomes more apparent. In fact, the calculation of the strength μ is similar to that of the net force in two dimensions. Instead of the forces, the hydrophobic values are applied here. In this study, *N* was set to 10 and the original hydrophobic scale proposed by Janin [18] was utilized. It is worth noting that the notion of hydrophobic moment is universal regardless of which hydrophobic scale is used [17]. In addition, to represent the optimal amphipathic site, the maximum amphipathicity was then selected for each AMP.

### 4.3. AMP Net Charge

AMP net charge is the difference between the count of the positively charged residues and that of the negatively charged residues of an AMP.

## Figures and Tables

**Table 1:** Significant antimicrobial activities of antimicrobial peptides (AMPs) in relation to amphipathicity and net charge.

Activities	N	Amphipathicity			Net Charge		
		Median	Range	*p*-Value	Median	Range	*p*-Value
P+ ${}^{a}$							
Yes	778	0.599	0.118∼1.219	4.05 × 10^−5^	3	−6∼14	3.35 × 10^−7^
No	142	0.540	0.072∼0.981		2	−7∼13	
N+ ${}^{a}$							
Yes	704	0.615	0.118∼1.219	4.07 × 10^−18^	4	−6∼14	9.31 × 10^−17^
No	216	0.485	0.072∼0.963		2	−7∼13	
F+ ${}^{a}$							
Yes	414	0.603	0.072∼1.189	9.11 × 10^−3^	4	−7∼14	1.29 × 10^−4^
No	506	0.572	0.118∼1.219		3	−6∼13	
P+N+ ${}^{a}$							
Yes	642	0.616	0.118∼1.219	7.84 × 10^−17^	4	−6∼14	3.59 × 10^−16^
No	278	0.504	0.072∼0.981		2	−7∼13	
P+F+ ${}^{a}$							
Yes	346	0.618	0.180∼1.189	6.22 × 10^−6^	4	−3∼14	5.12 × 10^−10^
No	574	0.566	0.072∼1.219		3	−7∼13	
N+F+ ${}^{a}$							
Yes	321	0.625	0.180∼1.189	1.39 × 10^−10^	4	−3∼14	4.71 × 10^−15^
No	599	0.558	0.072∼1.219		3	−7∼13	
P+N+F+ ${}^{a}$							
Yes	313	0.626	0.180∼1.189	8.96 × 10^−11^	4	−3∼14	1.58 × 10^−14^
No	607	0.558	0.072∼1.219		3	−7∼13	
P+ vs. N+F+ ${}^{b}$							
P+ (Yes)	778	0.599	0.118∼1.219	2.63 × 10^−4^	3	−6∼14	9.76 × 10^−6^
N+F+ (Yes)	321	0.625	0.180∼1.189		4	−3∼14	
P+ vs. P+N+F+ ${}^{b}$							
P+ (Yes)	778	0.599	0.118∼1.219	1.82 × 10^−4^	3	−6∼14	1.17 × 10^−5^
P+N+F+ (Yes)	313	0.626	0.180∼1.189		4	−3∼14	

Abbreviations: P+, anti-gram-positive bacteria; N+, anti-gram-negative bacteria; F+, anti-fungi. Gray shading: pass the Bonferroni test (α = 0.01). ${}^{a}$: 31 hypotheses were tested; ${}^{b}$: 21 hypotheses derived from any two out of seven significant relationships were tested.

## Abbreviations

The following abbreviations are used in this manuscript:

AMP	Anti-microbial peptide

## References

[1] Lauth X., Shike H., Burns J.C., Westerman M.E., Ostland V.E., Carlberg J.M., Van Olst J.C., Nizet V., Taylor S.W., Shimizu C. (2002). Discovery and characterization of two isoforms of moronecidin, a novel antimicrobial peptide from hybrid striped bass. J. Biol. Chem..

[2] Wachinger M., Kleinschmidt A., Winder D., von Pechmann N., Ludvigsen A., Neumann M., Holle R., Salmons B., Erfle V., Brack-Werner R. (1998). Antimicrobial peptides melittin and cecropin inhibit replication of human immunodeficiency virus 1 by suppressing viral gene expression. J. Gen. Virol..

[3] Wang H., Ng T.B. (2006). Ganodermin, an antifungal protein from fruiting bodies of the medicinal mushroom Ganoderma lucidum. Peptides.

[4] Choi S.J., Parent R., Guillaume C., Deregnaucourt C., Delarbre C., Ojcius D.M., Montagne J.J., Celerier M.L., Phelipot A., Amiche M. (2004). Isolation and characterization of Psalmopeotoxin I and II: Two novel antimalarial peptides from the venom of the tarantula Psalmopoeus cambridgei. FEBS Lett..

[5] Jabrane A., Sabri A., Compere P., Jacques P., Vandenberghe I., Van Beeumen J., Thonart P. (2002). Characterization of serracin P, a phage-tail-like bacteriocin, and its activity against Erwinia amylovora, the fire blight pathogen. Appl. Environ. Microbiol..

[6] Tossi A., Sandri L. (2002). Molecular diversity in gene-encoded, cationic antimicrobial polypeptides. Curr. Pharm. Des..

[7] Lee H.T., Lee C.C., Yang J.R., Lai J.Z., Chang K.Y. (2015). A large-scale structural classification of antimicrobial peptides. Biomed. Res. Int..

[8] Wang G., Li X., Wang Z. (2016). APD3: The antimicrobial peptide database as a tool for research and education. Nucleic Acids Res..

[9] Waghu F.H., Barai R.S., Gurung P., Idicula-Thomas S. (2016). CAMPR3: A database on sequences, structures and signatures of antimicrobial peptides. Nucleic Acids Res..

[10] Takahashi D., Shukla S.K., Prakash O., Zhang G. (2010). Structural determinants of host defense peptides for antimicrobial activity and target cell selectivity. Biochimie.

[11] Zhu X., Dong N., Wang Z., Ma Z., Zhang I., Ma Q., Shan A. (2014). Design of imperfectly amphipathic alpha-helical antimicrobial peptides with enhanced cell selectivity. Acta Biomater..

[12] Wang G. (2010). Antimicrobial Peptides: Discovery, Design and Novel Therapeutic Strategies.

[13] Koren E., Torchilin V.P. (2012). Cell-penetrating peptides: Breaking through to the other side. Trends Mol. Med..

[14] Brogden K.A. (2005). Antimicrobial peptides: Pore formers or metabolic inhibitors in bacteria?. Nat. Rev. Microbiol..

[15] Chang K.Y., Lin T.P., Shih L.Y., Wang C.K. (2015). Analysis and prediction of the critical regions of antimicrobial peptides based on conditional random fields. PLoS ONE.

[16] Huang Y., Niu B., Gao Y., Fu L., Li W. (2010). CD-HIT Suite: A web server for clustering and comparing biological sequences. Bioinformatics.

[17] Eisenberg D., Weiss R.M., Terwilliger T.C. (1982). The helical hydrophobic moment: A measure of the amphiphilicity of a helix. Nature.

[18] Janin J. (1979). Surface and inside volumes in globular proteins. Nature.

